# Supplementary material for: A single ‘weight-lifting’ game covers all kinds of games
Source: R Soc Open Sci. 2019 Nov 20;6(11):191602. doi: 10.1098/rsos.191602 (PMC6894607; doi:10.1098/rsos.191602)
Supplement: Supplementary Information [file rsos191602supp1.pdf]

Supplementary material:

A single 'weight-lifting' game covers all kinds of games by

Tatsuki Yamamoto, Hiromu Ito, Momoka Nii, Takuya Okabe, Satoru Morita and Jin Yoshimura

Equivalence of pairwise C-D games and the weight-lifting game

In this proof, we first show that (1) all the C-D games are covered as special cases of the weight-lifting game, and then that (2) the weight-lifting game corresponds to a certain case of the C-D games. In terms of players' strategies, cooperation (C) and defection (D), the payoff matrix of pairwise games are given as follows:

$$A = \begin{matrix} & \mathrm{C} & \mathrm{D} \\ \mathrm{C} & \multicolumn{2}{c}{\begin{pmatrix} \mathrm{R} & \mathrm{S} \\ \mathrm{T} & \mathrm{P} \end{pmatrix}} \\ \mathrm{D} & & \end{matrix}.$$

The C-D games satisfy three conditions $\mathrm{R} \geq \mathrm{S}$, $\mathrm{T} \geq \mathrm{P}$ and $\mathrm{T} \geq \mathrm{S}$.

(1)

(i) If $\mathrm{T} \neq \mathrm{P}$, the game is transformed into an equivalent game with the following matrix elements:

$$A = \begin{array}{c} \\ \mathrm{C} \\ \mathrm{D} \end{array} \begin{array}{c} \mathrm{C} \quad \mathrm{D} \\ \begin{pmatrix} r & s \\ 1 & 0 \end{pmatrix} \end{array},$$

where $r \geq s$ and $s \leq 1$. On the other hand, the payoff matrix of the weight-lifting game is given by

$$A = \begin{array}{c} \\ \mathrm{C} \\ \mathrm{D} \end{array} \begin{array}{c} \mathrm{C} \quad \mathrm{D} \\ \begin{pmatrix} bp_2 - c & bp_1 - c \\ bp_1 & bp_0 \end{pmatrix} \end{array},$$

with $p_0 \leq p_1$, $p_1 \leq p_2$ and $c \geq 0$. For $p_1 \neq p_0$, the last matrix is equivalently put into

$$A = \begin{array}{c} \\ \mathrm{C} \\ \mathrm{D} \end{array} \begin{array}{c} \mathrm{C} \quad \mathrm{D} \\ \begin{pmatrix} (b(p_2 - p_0) - c)/b/(p_1 - p_0) & (b(p_1 - p_0) - c)/b/(p_1 - p_0) \\ 1 & 0 \end{pmatrix} \end{array}.$$

Comparing the two games, we have $r = (b(p_2 - p_0) - c)/b/(p_1 - p_0) = p_2/(p_1 - p_0) - p_0/(p_1 - p_0) - c/b/(p_1 - p_0)$ and $s = (b(p_1 - p_0) - c)/b/(p_1 - p_0) = 1 - c/b/(p_1 - p_0)$, so that $c/b = (1 - s)(p_1 - p_0) \geq 0$ and $r - s = (p_2 - p_0)/(p_1 - p_0) - 1 \geq 0$. For instance, one may take $p_0 = 0$, $p_2 = 1$, $p_1 = 1/(r - s + 1)$ and $c/b = (1 - s)/(r - s + 1) > 0$, for which $0 < p_1 < 1$, i.e., $p_1 \neq p_0$.

(ii) If $\mathrm{T} = \mathrm{P}$, the game matrix is transformed into

$$A = \begin{array}{c} \\ \mathrm{C} \\ \mathrm{D} \end{array} \begin{array}{c} \mathrm{C} \quad \mathrm{D} \\ \begin{pmatrix} r & s \\ 0 & 0 \end{pmatrix} \end{array},$$

where $r \geq s$ and $s \leq 0$. This is compared with the weight-lifting game with $p_0 = p_1$, which is equivalent to

$$A = \begin{array}{c} \\ \mathrm{C} \\ \mathrm{D} \end{array} \begin{array}{c} \mathrm{C} \quad \mathrm{D} \\ \begin{pmatrix} b(p_2 - p_0) - c & -c \\ 0 & 0 \end{pmatrix} \end{array}.$$

For $r = b(p_2 - p_0) - c$ and $s = -c$, we obtain $s = -c \leq 0$ and $r - s = b(p_2 - p_0) \geq 0$. For

instance, $c/b = \ -s\ /(r-s) \geq 0$ for $p_0 = 0,\ p_1 = 0$ and $p_2 = 1$.
Thus, all pairwise games are a special case of the weight-lifting game.

(2)
The method of proof by contradiction is used by assuming the opposite of the conditions R ≥ S, T ≥ P, and T ≥ S. Here the special case $b = r + f = 0$ is excluded because this is a trivial game of

$$\mathrm{A} = \begin{array}{c} \\ \mathrm{C} \\ \mathrm{D} \end{array} \begin{array}{c} \mathrm{C} \quad \mathrm{D} \\ \begin{pmatrix} -c & -c \\ 0 & 0 \end{pmatrix} \end{array}.$$

(i) Case R < S
If the condition R ≥ S is not satisfied,

$$\mathrm{R} < \mathrm{S} \Leftrightarrow (r-c)p_2 - (f+c)(1-p_2) < (r-c)p_1 - (f+c)(1-p_1)$$
$$\Leftrightarrow rp_2 - cp_2 + fp_2 - f - c + cp_2 < rp_1 - cp_1 + fp_1 - f - c + cp_1$$
$$\Leftrightarrow rp_2 + fp_2 < rp_1 + fp_1$$
$$\Leftrightarrow (r+f)p_2 < (r+f)p_1.$$

Therefore, we obtain $p_2 < p_1$ because $r + f > 0$. This contradicts the assumption $p_1 \leq p_2$. Thus, R ≥ S for the weight-lifting game.
(ii) Case T < P.
If the condition T ≥ P is not satisfied,

$$\mathrm{T} < \mathrm{P} \Leftrightarrow rp_1 - f(1-p_1) \ < rp_0 - f(1-p_0)$$
$$\Leftrightarrow rp_1 - f + fp_1 < rp_0 - f + fp_0$$
$$\Leftrightarrow rp_1 + fp_1 < rp_0 + fp_0$$
$$\Leftrightarrow (r+f)p_1 < (r+f)p_0$$

Therefore, $p_1 < p_0$ owing to $r + f > 0$. Since this contradicts $p_0 \leq p_1$, the condition T ≥ P is satisfied.
(iii) Case T < S
If the condition T ≥ S is not satisfied,

$$\mathrm{T} < \mathrm{S} \Leftrightarrow rp_1 - f(1-p_1) < (r-c)p_1 - (f+c)(1-p_1)$$
$$\Leftrightarrow rp_1 - f + fp_1 < rp_1 - cp_1 - f + fp_1 - c + cp_1$$
$$\Leftrightarrow c < 0.$$

This contradicts $c \geq 0$. Thus, the condition T ≥ S holds true. To sum up, the weight-lifting game is a pairwise game with R ≥ S, T ≥ P and T ≥ S.
